# Exploring the drivers of reef island shoreline change using machine learning models

**DOI:** 10.1038/s41598-025-00136-w

**Published:** 2025-05-14

**Authors:** Meghna Sengupta, Murray R. Ford, Paul S. Kench, George L.W. Perry

**Affiliations:** 1 Leibniz Centre for Tropical Marine Research (ZMT), Bremen, Germany; 2 School of Environment, The University of Auckland, Auckland, New Zealand; 3 Department of Geography, National University of Singapore, Singapore, Singapore

**Keywords:** Coral reef islands, Atolls, Shoreline change, Pacific ocean, Machine learning, Climate change, Geomorphology, Environmental impact

## Abstract

Empirical records of reef island shoreline change show magnitude and styles of island change are highly variable over various spatio-temporal scales. However, the attribution of processes as drivers of observed change is poorly resolved. In this study, we develop machine-learning models to explore the drivers of shoreline and positional change of island footprints using multi-decadal records spanning the western-central Pacific. Our models identify a set of ‘important’ predictors, notably a combination of oceanographic, climatic, and local-scale morphological properties of islands and reef platforms. Additionally, we use the models to examine the interactions between these predictors. Results offer the first machine-learning models for reef island physical change, and highlight the complex relationships between a range of controls. While sea-level rise is considered a uniform threat across all islands, our results illustrate that the direct erosional response to high sea-level rise rates was attenuated in settings of ‘positive’ local-scale properties, such as broader reef platforms, and/or high vegetation density; underscoring the necessity for nuanced adaptation strategies that acknowledge local-scale variabilities. Results have implications for understanding attribution, developing vulnerability indices for small islands, and lay the groundwork for projections of island change as effects of climate change intensify over the coming decades.

**Supplementary Information:**

The online version contains supplementary material available at 10.1038/s41598-025-00136-w.

## Introduction

Impacts of anthropogenic climate change, particularly sea-level rise and changes in wave regime, are expected to destabilise low-lying coral reef islands over the 21st century^1–3^. Atoll nations face an increasingly uncertain future, with the IPCC’s Sixth Assessment Report projecting global mean sea-levels to rise by 0.28 to 0.55 m under SSP1-1.9, and 0.63 to 1.01 m under SSP5-8.5 by the end of the century^4,5^. Consequently, there have been increasing global efforts towards the establishment of comprehensive empirical baseline datasets on coastal change and dynamics across small islands^6–8^. In addition, the critical need to identify the primary drivers of these changes and the complex interactions between them, to inform and optimise adaptation strategies and decision-making has been emphasised by recent studies^8,9^.While a growing number of studies have taken an observational approach to record island change over periods of sea-level rise, define styles of island change, and record event-based responses, the attribution of processes as drivers of island change remains poorly resolved^6,7^.

Existing studies of island change, particularly those that use remote sensing data with transect-scale analysis^10–12^, offer a substantially large, valuable record of shoreline changes at fine spatial scales, and compilation of such data provides the opportunity to quantify and examine patterns of island change and explore relationships between response and key controls through statistical analyses. However, such efforts towards linking island change to processes have been limited to traditional statistical models, such as linear regressions^13,14^, which due to their pre-assumed structure, are often unable to identify meaningful patterns in large and complex datasets. To identify structures in high dimensional data, Machine Learning (ML) models have increasingly gained popularity over the past decades^15–18^. Within reef island research, the use of ML models has largely been limited to raster-based analysis for the classification of geomorphic zones and monitoring coral reef health^19–21^, and they have not been used to model reef island shoreline change. In a recent study, Sengupta et al. (2023)^14^ analysed a large-scale, high-resolution shoreline change record of 568 islands from 42 atolls in the Pacific alongside 25 potential predictors quantified using remote sensing methods. These predictors included a range of regional-scale oceanographic and climatic characteristics such as sea-level rise, wave energy, and storms^22–24^ as well as local-scale characteristics of islands and their underlying reef platforms (e.g. reef width, island size, shape, vegetation density). Due to the geographical expanse of this dataset spanning the western to the central Pacific, the sample of islands cover a wide gradient of climatic and hydrodynamic setting as well as diverse morphological characteristics. However, using classical statistical methods of linear regression, no strong association was found between island change and individual variables. This study highlighted that the widely perceived notion of a linear relationship between shoreline change and individual process drivers, such as sea-level rise is overly simplistic, and there is a need for more robust models that can explore the complex relationships between a range of predictors and island physical change.

In this study, we use this dataset to develop a set of machine-learning models to address two objectives: (1) identifying a set of ‘important’ variables, which has direct implications for decision-making for further research, and the development of vulnerability indices, and (2) using exploratory models to examine the interactions between the identified ‘important’ variables, enabling an investigation into the substantial heterogeneity and non-linearity in the relationships between a range of processes driving island change.

## Results

### Controls on island shoreline change

**Fig. 1:**
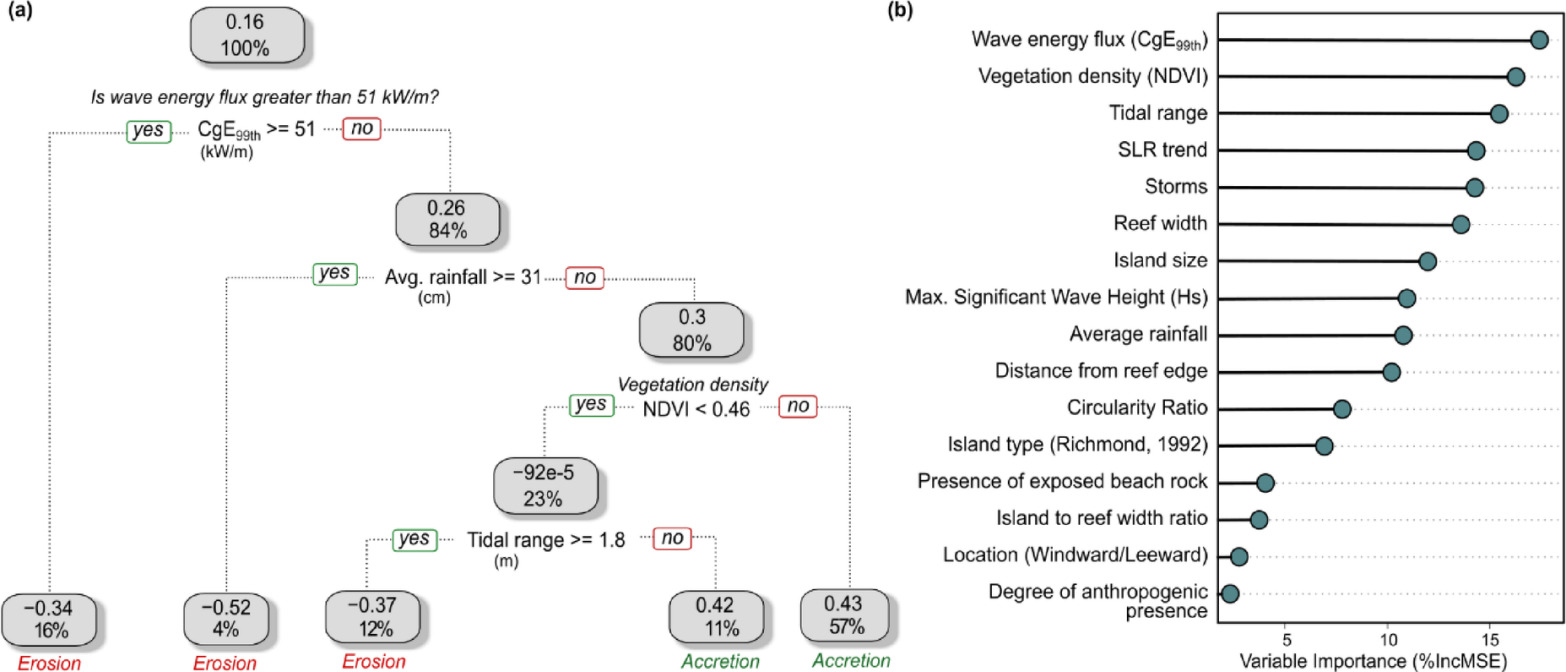
(**a**) Pruned CART model for island shoreline change rate (m/decade) as response variable (**b**) Variable importance rankings from the Random Forest model.

The CART model provides a heuristic graphical representation where the leaf nodes indicate the average shoreline change rate and the percentage of the sample dataset in each terminal node (Fig. 1a). The Random Forest model is used to illustrate the rankings of variable importance based on the percentage increase in Mean Square Error (%IncMSE) of the model when the values of a particular variable are randomly permuted while all other variables are left unchanged. Therefore, a higher %IncMSE indicates higher importance of the variable (Fig. 1b). Both the CART and the Random Forest models identified a combination of climatic, oceanographic, and local-scale properties as important determinants of recorded change, including the 99th percentile of wave energy flux (CgE_99 th_), vegetation density [indicated by Normalised Difference Vegetation Index (NDVI)], and tidal range (Fig. 1).

### Controls on rate of island migration

**Fig. 2:**
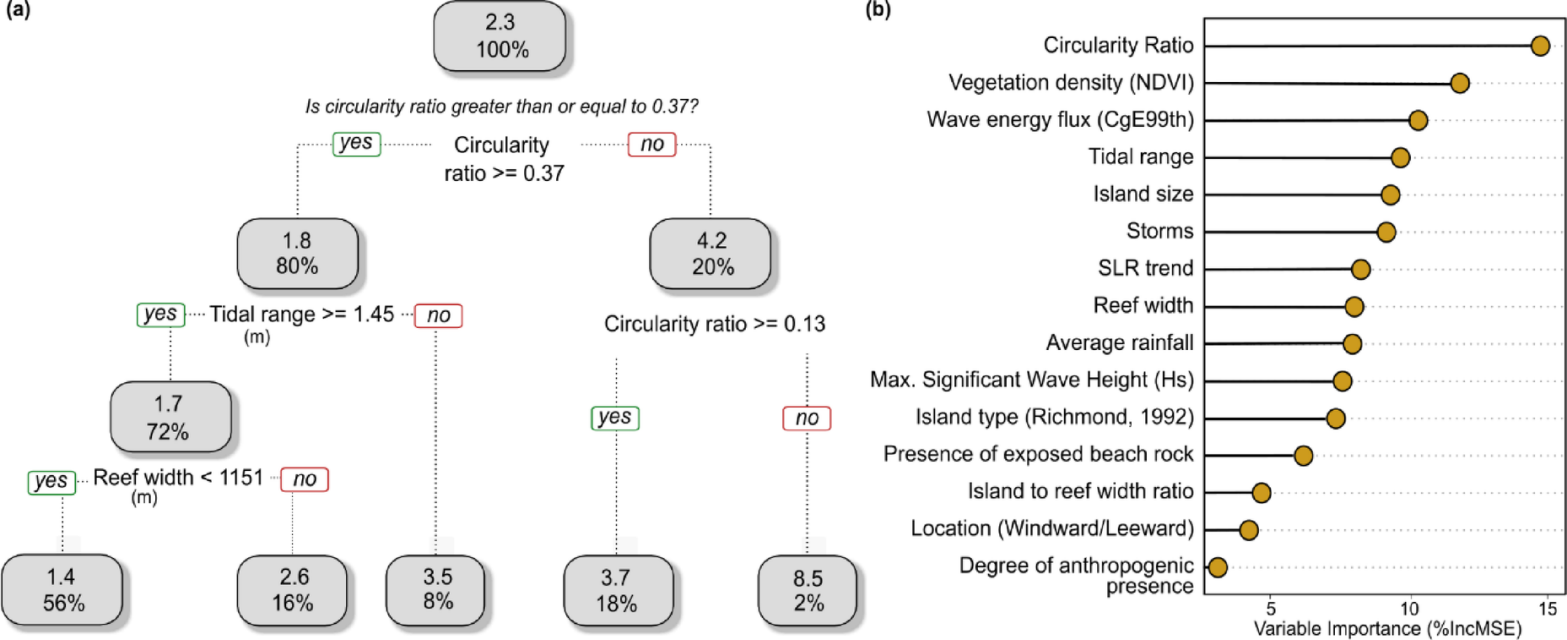
(**a**) Pruned CART model for migration rate (m/decade) as response variable, (**b**) Variable importance rankings from the Random Forest model.

Island migration is the net outcome of local-scale shoreline changes around the entire island and is recorded as the Euclidean distance between temporally consecutive island centroids normalised by time. It provides a measure of the long-term trajectory of island positional change on the reef platform and can be a key metric for adaptation decision-making. Both the CART and RF models show migration rate is predominantly controlled by island shape, vegetation density, tidal range, and the width of the underlying reef platform, with circularity ratio (indicator of island shape) having the highest importance (Fig. 2). The largest average rate of migration occurs across elongate or irregular islands (circularity ratio < 0.13), while the smallest rates of migration are amongst those with a comparatively more circular shape (circularity ratio > 0.37).

### Interactions between important controls on island change

**Fig. 3:**
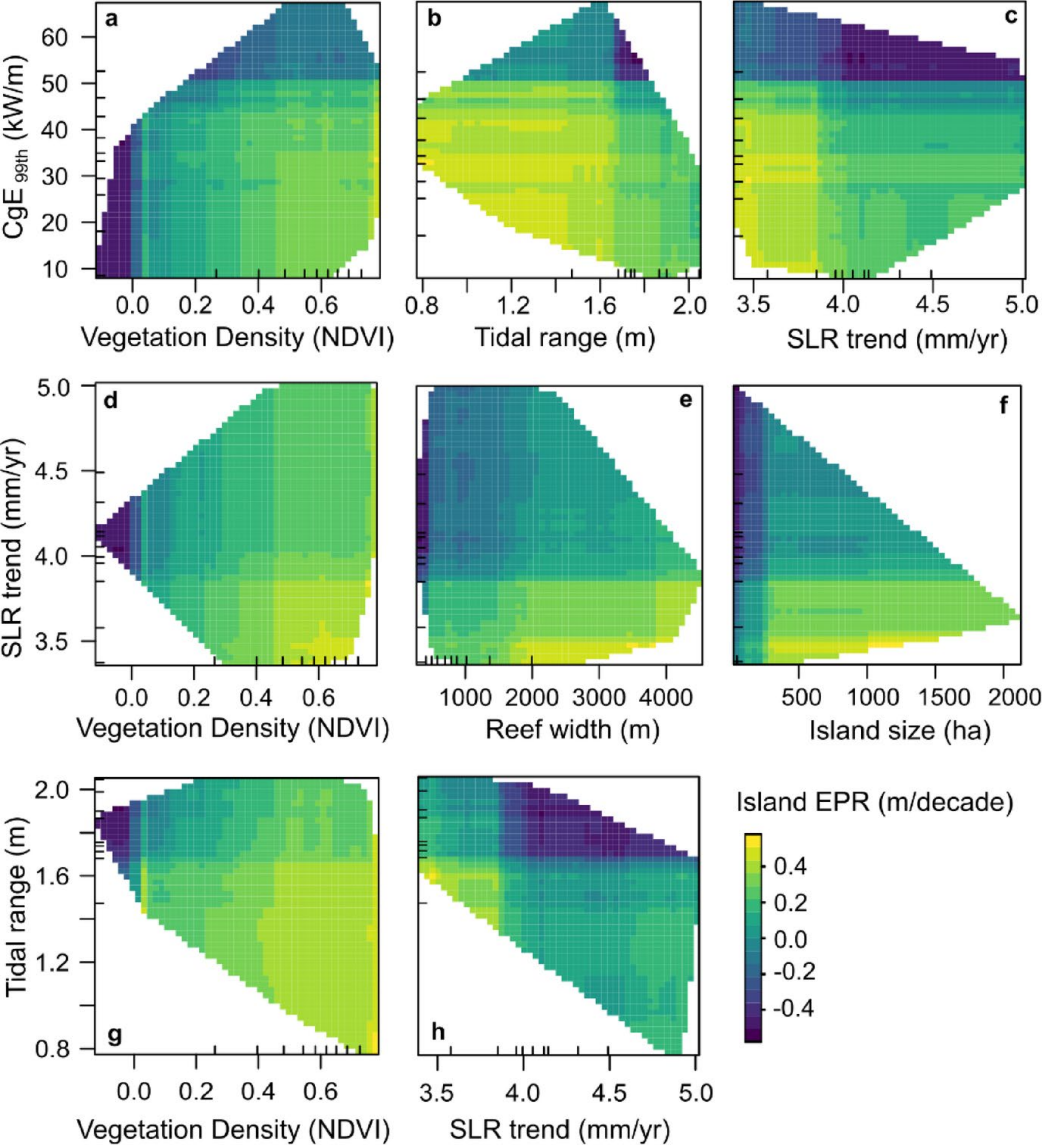
Partial dependency plots showing interactions between the most relevant variables of island shoreline change rate, Island EPR (m/decade). Colour gradient indicates high erosion (dark blue) to high accretion (yellow). The ticks on the x and y-axis are rug plots that show the distribution of values of the predictor variables.

Results from the models highlight the role of a set of ‘important’ variables in defining the variability of rates of island change within our dataset. Of note, these variables include both regional-scale climatic and oceanographic setting, as well as local-scale morphometric properties of islands and reefs. Partial dependence plots from the Random Forest model illustrate the interactions between these relevant variables controlling island shoreline change (Fig. 3) and highlight their non-linear associations. Some of the lowest shoreline change rates occur on islands with very low vegetation density (low NDVI scores), reflecting the typically erosional trend across sparsely vegetated islands (Fig. 3a, d, g) with respect to wave energy, SLR, and tidal range. Interactions between SLR and other important local-scale predictors highlight the diverse responses of islands across distinct settings (Fig. 3). While accretion is predominant on islands on broad reef platforms, erosion is observed across islands on narrow reef platforms and exposed to SLR rates of > 4.0 mm/year (Fig. 3e). Further examination of interactions between SLR rate and tidal range shows a sharp break indicating a threshold response, where shorelines are typically erosional when exposed to larger tidal ranges (> 1.6 m) as well as larger SLR rates (> 4.0 mm/year) (Fig. 3h).

**Fig. 4:**
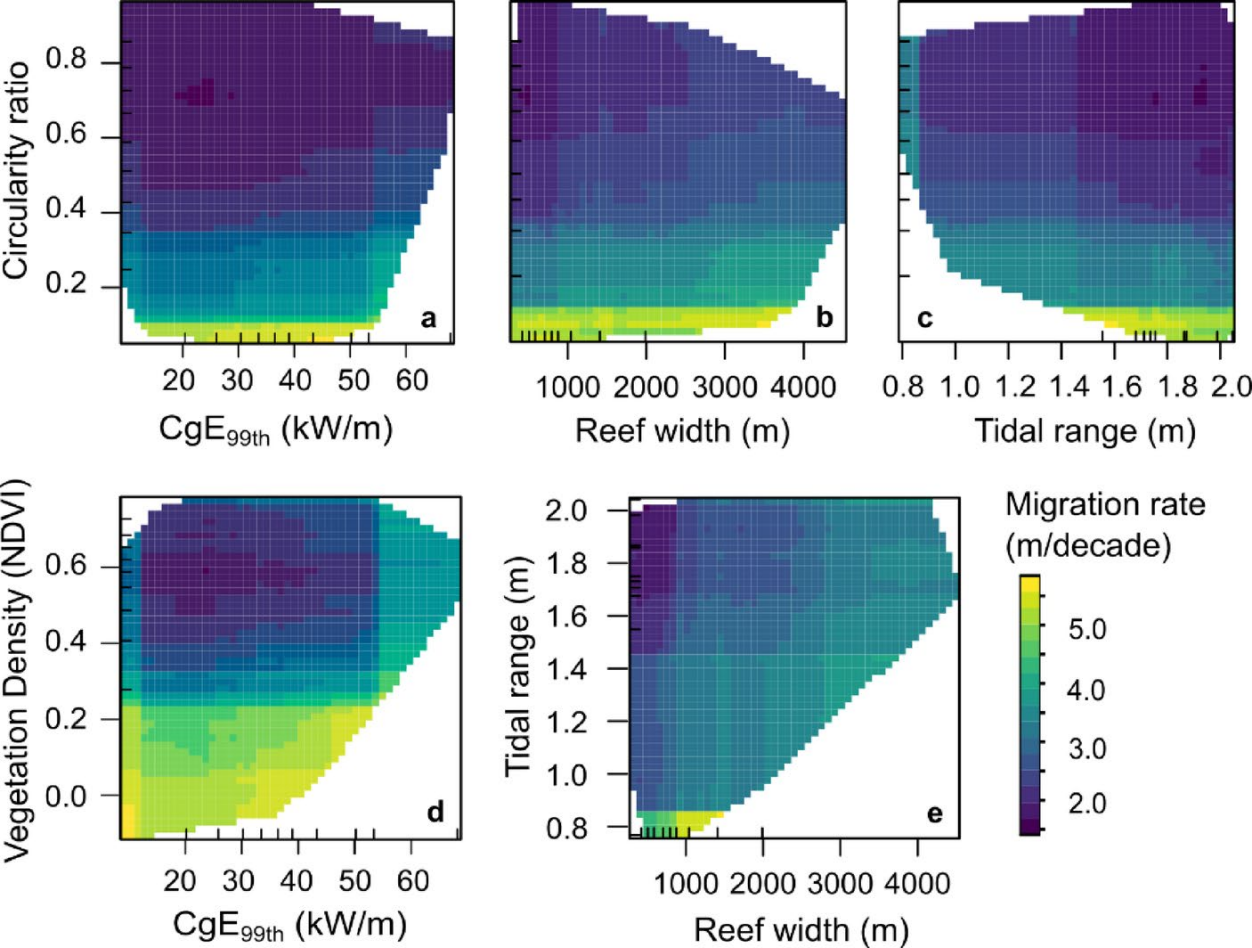
Partial dependency plots showing interactions between the most relevant predictors of rate of migration of reef islands. Colour gradient indicates low (dark blue) to high (yellow) rates of migration. The ticks on the x and y-axis are rug plots that show the distribution of values of the predictor variables.

Both ML models identify island shape (defined by circularity ratio) as the most important control on rate of migration, followed by vegetation density, wave energy flux as well as reef width (Fig. 2). Partial dependency plots for rate of migration reveal high migration rates typically occur on islands with low circularity ratio – i.e. islands that are largely elongate or irregular in shape and are more susceptible to shifts in alongshore drift processes. A gradient of increasing migration rate can be observed across increasing or decreasing gradients of various predictors (Fig. 4). On the interaction plot between circularity ratio and reef width (Fig. 4b), the increase in migration rate is evident with increasing reef width and decreasing circularity ratio. The lowest rates are concentrated across islands on narrow reef platforms and high circularity ratio (near-circular islands); while some of the highest rates are found across broad reef platforms and low circularity ratio (elongate/irregular islands). The interaction between tidal range and reef width show a gradient of increasing migration rate with increasing reef platform width across settings of relatively larger tidal range, i.e. > 1.6 m (Fig. 4e).

## Discussion

The use of a high-resolution multidecadal shoreline change record of coral reef islands spanning the western to central Pacific Ocean and data capturing a range of potential controls, describing various oceanographic, climatic and local-scale morphometric parameters, provided the opportunity to explore relationships between a range of processes and island response since the mid-20th century. Using two ML algorithms, CART and Random Forest, a set of models were developed that identified key controls on rates of island shoreline change and rates of island migration. Furthermore, results from these models were used to illustrate and examine the interactions between the important predictors.

### Is sea-level rise a good predictor of the variability in magnitude of island change?

Results from the ML models highlight SLR as one of the ten most important predictors of island change along with a range of other variables, including wave energy, tidal range and vegetation density, that were relevant in explaining the variability in magnitude of shoreline change rates across the study islands since the mid-20th century. Exploring variable interactions in the Random Forest model revealed distinct shoreline changes along a gradient of SLR with respect to these other variables – such as erosional trends on islands exposed to higher rates of SLR (> 4.0 mm/year) coupled with higher tidal range, higher wave energy, lower density of vegetation, or islands perched on narrow reef platforms. Collectively, these results suggest that while SLR is a factor that can influence physical island change, SLR has not driven a uniform response across the study islands over the past half-century, and a range of other variables, including regional-scale wave climate and island’s local-scale properties also influence the magnitude and variability of island change across the Pacific. We caution that these results are entirely based on observations in recent decades where the range of SLR observed were between 3.19 mm/year and 5.03 mm/year in our dataset^23,25^, and the interactions with other variables (such as local water depth that control net wave energy interacting with shorelines) may change with large increase in rates of SLR such as projected under SSP5-8.5^4^. Further research using high-frequency shoreline change records, particularly in the altimetry era (1993-present), could be helpful in tracking any sea-level rise signals reflected in island shoreline response within atoll settings, and provide the opportunity to generate change projections under various RCP/SSP scenarios.

A key inference that can be drawn from the outcomes of our study is that the direct erosional response of shorelines to higher rates of sea-level rise was attenuated in settings of ‘positive’ local-scale properties – i.e. settings of small tidal range, naturally low energy settings, islands perched on broader reef platforms, and/or islands with high vegetation density. Therefore, we highlight the need for adaptation strategies to incorporate this inherent variability when defining potential tipping points for adaptation interventions, and developing strategies that are catered exclusively based on the local hydrodynamic and morphological settings across various atolls or island groups.

### Importance of reef and island morphometrics

While oceanographic variables such as sea-level rise and wave climate are often linked to island changes^11,26,27^, the influence of local-scale morphological parameters in driving shoreline changes remains a challenge to quantify^28^. The outcomes of the ML models highlight the importance of local-scale controls. Morphological properties such as reef width and circularity ratio were identified as key controls on island change (Figs. 1 and 2). While water depth and wave energy (including high energy events) are important factors in generating and transporting sediments, the morphological characteristics of the reef platform have been shown to regulate sediment transport pathways and thus patterns of accretion or erosion along island shorelines^29–31^.

It is noteworthy that island shape (quantified by circularity ratio) emerges as a key control of the rate of island migration. Wave transformation around a near-circular island has been shown to differ from that around an elongate island^30,32^. Islands on elongate platforms are influenced by wave refraction processes that are predominantly fixed, and controlled by the higher angle curvature of the reef structure, leading to more oblique angles of wave approach at island shorelines, driving alongshore currents. This is a stable relationship, however, if this should change then the alongshore current pattern may change more permanently driving redistribution of sediments. On circular islands, the wave processes and shoreline sediment fluxes are very sensitive to changes in wave approach and respond continuously. Therefore, keeping the deposition centre constant, resulting in a near-stability of island shape and position on the reef platform. These contrasting behaviours are evident in the results of our study. ML models show migration rates of near-circular islands are notably lower than those of elongate/irregular islands. Furthermore, reef width is identified as another important control that potentially determines the magnitude of migration for near-circular islands; large reef widths provide the accommodation space required for higher rates of migration, whereas, for islands on narrow reef platforms, migration is largely restricted, and sediments are likely lost off the edge of the platform. Island mobility is a key indicator of island response to changing environmental conditions^8^, and the magnitude of migration has implications for predicting the long-term trajectory of an island. Our results underscore the necessity of including reef morphometrics in island vulnerability indices. It is important to note that, albeit at a different time-scale than islands, reef morphology is also susceptible to change in response to changing environmental conditions and declining reef health, leading to the loss of surface roughness and degradation of reef structures, increasing the likelihood of wave driven erosion and flooding^33–35^.

### Vegetation density and its interactions with other predictors

Many studies have highlighted the role of vegetation in supporting the long-term physical persistence of reef islands by stabilising island sediments, providing provisions of improved island growth and enhancing adaptability to changing boundary conditions^36–39^. Our results are consistent with these studies and show that vegetation density, which is an island-specific characteristic, has played an important role in defining the patterns of island change observed across the Pacific. A trend of erosion across sparsely vegetated islands across the observed gradient of wave energy illustrates the susceptibility of such islands and reflects the sediment binding properties of island vegetation, indicating that islands with stable dense vegetation are less likely to undergo wave-driven erosion, though we note that events such as wave overtopping can temporarily reduce island vegetation density. While accretion is predominant on densely vegetated islands, there is a trend of increase in the magnitude of accretion with decreasing wave energy flux, decreasing tidal range, and decreasing rates of SLR, implying that an idealised setting for steady island growth is a densely vegetated island in a comparatively low energy setting with a small tidal range. This is consistent with studies that have explored sediment transport and depositional patterns in various tidal settings and have concluded that a small tidal range provides optimal conditions for sediment deposition and accretion^29,40^. In such environments, the limited vertical movement of water reduces the reworking of sediments, allowing finer materials to accumulate and contribute to island growth. These low-energy conditions promote the trapping of sediments, enhancing the potential for island accretion and resilience against sea-level rise^29,40,41^. We appreciate that this is also linked to relative water depth that is likely variable across islands and may influence the magnitude of wave driven geomorphic work occurring on shorelines. While sea-level rise is widely considered a primary metric in assessing island physical vulnerability^27,41–43^, our study underscores that vegetation density, local wave and tidal regime and reef morphometrics are key parameters that should be incorporated in future studies formulating risk assessments and vulnerability indices for atoll islands.

### Constraints and prospects for further efforts in modelling island change

Our study spans across a vast longitudinal and latitudinal extent, and the relatively large sample of islands constrains the use of field-sampled data. Consequently, the variables used in this study are limited to those that can be extracted from remote sensing and/or model data. This provides a valuable test of the use of such datasets for large-scale studies and highlights the use and performance of open source data in developing such models. However, we acknowledge that some characteristics of islands could not be explicitly quantified using this approach. For example, sediment calibre is closely linked to an island’s geomorphic maintenance, and there is ample evidence of the role of sediment productivity and grain size in island building, elevation, and location of deposition on the reef platform^44–47^. Nevertheless, several predictors used in this study are coupled with some of these factors and may act as surrogates – such as by classifying islands as windward or leeward or capturing ‘island type’ based on Richmond (1992)^48^. The lack of high-resolution Digital Elevation Models for the Pacific also limits the quantification of volumetric changes on the study islands, however, planform shoreline progradation or recession can be assumed to indicate either sufficient availability of sediments and mechanisms driving these sediments onto island systems promoting island shoreline accretion or a net loss in sediments resulting in the recession of island shorelines. We emphasise that efforts to produce high-resolution remote sensing and model data that can quantify local-scale wave characteristics, incorporate impacts of high-energy events, sediment calibre, relative water depth on the reef platforms and changes in island elevation are critical, and the availability of such datasets would significantly refine island change models such as those presented here. The use of Explainable Artificial Intelligence may also provide prospects for exploring island specific models, that offer further insights into inter-island variabilities in morphological responses^49^. The availability of chronostratigraphic data particularly exploring the complex role of storms, field measurements of highly resolved wave characteristics across island shorelines, vegetation type, reef health, sediment grade and composition will be invaluable for such island-specific models.

### Implications for understanding attribution and projections of island change

Existing studies of reef island change provide an empirical record of island dynamics over periods of sea-level rise and local-scale evidence of attribution in response to event-based drivers of change^6,7^. In this study, we used published records of multidecadal shoreline changes of islands spanning an expansive longitudinal and latitudinal extent from the Federated States of Micronesia, Marshall Islands, Kiribati and Tuvalu, covering a broad gradient of sea-level rise rates, storm frequency, wave climate, as well as diverse local-scale morphometric characteristics of islands and their underlying reef platforms. Using ML-based models, our study provides the opportunity to explore the key controls on island change within the past half-century and examine their interactions. The variability in the magnitude of island change is explained by a combination of predictors that include both regional-scale oceanographic and climatic properties as well as local-scale characteristics of islands and reef platforms, and likely several other factors such as sediment grade and relative water depth not able to be accounted for in this study. Although the rate of sea-level rise does not appear to have driven a uniform response across the study islands, model results provide insights into patterns of island change as a function of a range of variables, including SLR, wave energy, tidal range, island shape, vegetation density and width of the reef platform, and highlights the interactions between these regional climatic drivers and local-scale island and reef properties.

Collectively, the outcomes of this study highlight the importance of incorporating a range of variables in reef island change models. Identifying important controls is instructive in isolating variables that should be scrutinised in climate models and provides an incentive to move towards a holistic approach in understanding island stability and vulnerability in the context of anthropogenic climate change. As climate-related pressures intensify, there is a need to use methodologies that are robust, flexible, and have the ability to handle large and complex datasets. Our results provide the first set of such models in the field of reef island research and lay the groundwork for future efforts in modelling projections of island change in response to changes in sea-levels, and wave climate over the coming decades. Model outcomes, like those presented in this study, can inform the development and refinement of vulnerability indices^50,51^ for island nations and provide the impetus to develop more nuanced adaptation strategies that appreciate the variability in local-scale conditions across small islands.

## Methods

### Island change data and predictor variables

This study uses recently published records of island change from 42 atolls spanning the western-central Pacific Ocean, from the Federated States of Micronesia, Marshall Islands, Kiribati and Tuvalu, and 25 potential predictor variables quantified at island-scale^8,14^. This dataset was compiled by analysing high-resolution satellite imagery and historical aerial photographs, manually digitising shorelines based on an edge-of-vegetation proxy, and a transect-based multidecadal shoreline change analysis performed using the Digital Shoreline Analysis System^52^. Change statistics were reported as island-averaged rates of shoreline change [Island End Point Rate (EPR)] and positional changes of island footprints (Migration rate). Supplementary Fig. 1 illustrates a summary of this island change data. The predictor variables included a range of climatic and oceanographic parameters, where values were extracted from various sources such as altimetry records^23,25^, wave hindcasts^22^, and storm records^24^. Additionally, local-scale properties of islands and their underlying reef platforms were interpreted from the high-resolution satellite imagery (see Sengupta et al. (2023)^14^ and Supplementary document for further details).

### Model development and evaluation

To identify and understand patterns in the data and explore the key predictors of reef island change, we developed two sets of machine-learning models, selected based on their capabilities in handling large datasets, incorporate both categorical and continuous predictor variables, and represent linear or nonlinear relationships and interactions. The first set of models uses a classification and regression tree (CART) algorithm, that demonstrate variable splits and provides a heuristic understanding of the decision-making process^53^. The second set of models are Random Forest models^54^ that generate many CART models that perform as an ensemble and therefore offer higher accuracy, and are used to examine variable importance and their interactions. These models have been used extensively in fields such as ecology^16^, geomorphology^18^, and are well documented in providing insights into the identification and exploration of important predictors.

From the 25 variables in the dataset presented by Sengupta et al. (2023)^14^, 16 variables were used as predictors in the models to avoid model performance issues that arise due to collinearity between variables (Supplementary Table 1 provides a detailed list and summary of the predictors). This selection was made based on the threshold value of absolute correlation coefficient of r > |0.7| following Dormann (2013)^55^. After standard formatting of the database of islands, including encoding categorical variables as *factors* and removing rows with any *null* values, CART models were developed for island-averaged shoreline change rate (*n* = 476) and rate of migration (*n* = 436) using the *rpart *package in R^56^. Since the response variables are continuous, regression trees were generated, cross-validation was performed, and the trees were pruned based on a cost-complexity approach^57^. The *randomForest *package in R^58^ was used to develop the Random Forest models, and hyperparameter tuning was performed to optimise model performance. Each model generated 500 trees with 5 predictor variables tried at each split (Supplementary Fig. 2). Model performance metrics, i.e. coefficient of determination (*R.sq.*), mean absolute error (MAE) and root mean square error (RMSE) are presented in Supplementary Table 2. Variable importance rankings were examined from the Random Forest model using the *varImpPlot()* function. Additionally, the *randomForestExplainer *package^59^ was used to: (1) plot the distribution of minimum depth by passing the forest to the *min_depth_distribution()* function (Supplementary Fig. 3); (2) to generate multi-way importance plots showing the relationship between mean square error (MSE) and node purity for the most relevant variables by running the *multi_way_importance()* function (Supplementary Fig. 4). Finally, partial dependence plots were generated using the *partial()* function in the *pdp *package^60^ to explore the interactions between the relevant variables. All statistical tests, model development and evaluation were conducted using R (version 4.0.2)^61^; Supplementary Table 3 lists all packages used.

## Electronic supplementary material

Below is the link to the electronic supplementary material.

Supplementary Material 1

## Data Availability

The datasets used in this study are available from previously published articles, as detailed in the Methods section. All additional information supporting this work is provided in the manuscript and the supplementary document.
