# Supplementary material for: Exploring the drivers of reef island shoreline change using machine learning models
Source: Sci Rep. 2025 May 14;15:16735. doi: 10.1038/s41598-025-00136-w (PMC12078478; doi:10.1038/s41598-025-00136-w)
Supplement: Supplementary file 1 — Supplementary Material 1 [file 41598_2025_136_MOESM1_ESM.pdf]

Supplementary Information

Exploring the drivers of reef island shoreline change using machine learning models

Meghna Sengupta*[1,2], Murray R. Ford[2], Paul S. Kench[3], and George L.W. Perry[2]

[1]*Leibniz Centre for Tropical Marine Research (ZMT), Bremen, Germany*
[2]*School of Environment, The University of Auckland, Auckland, New Zealand*
[3]*Department of Geography, National University of Singapore, Singapore*
**Corresponding author e-mail: meghna.sengupta@lebiniz-zmt.de*

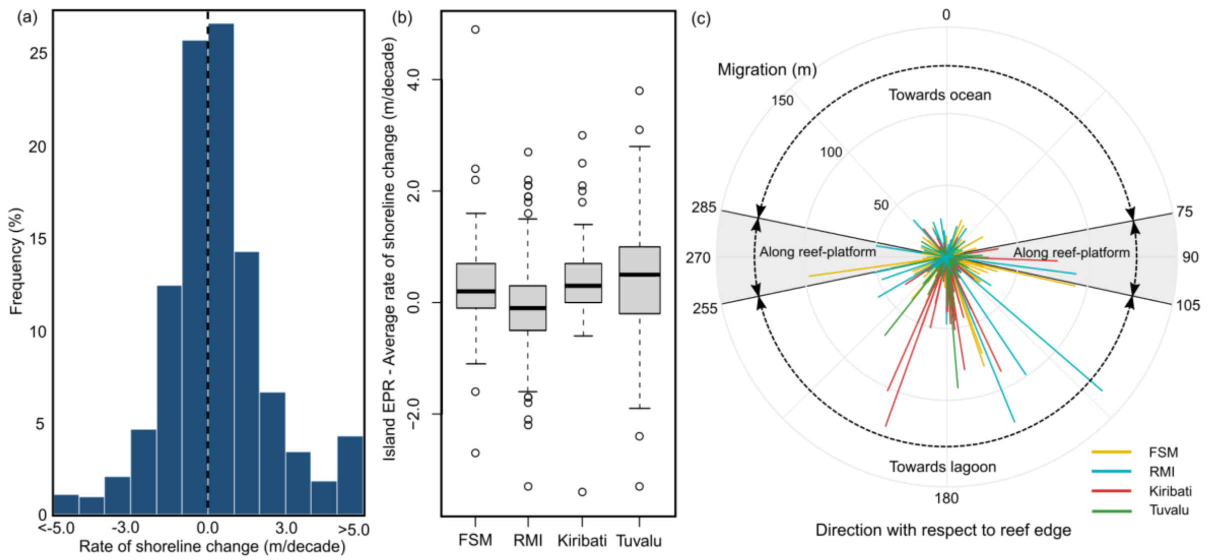

Supplementary Fig 1. Summary of shoreline changes across the Federated States of Micronesia, Republic of Marshall Islands, Kiribati and Tuvalu: (a) shoreline change rates across transects (frequency%), (b) island-averaged rates of shoreline change (c) magnitude of migration across islands (from Sengupta et al., 2023)

Supplementary Table 1. Summary of the potential controls (predictor variables) used in this study

Candidate/ Predictor Variables	Source/ Details	Method/data type	Data Summary (Min, mean, median, max.)
**Regional oceanographic and climatic characteristics**			
Sea Level Rise trend (mm/year)	AVISO (Ablain et al.,2018; Legeais et al., 2018)	NetCDF	3.19; 4.07; 4.01; 5.03
Hs max (m)	WaveWatchIII (Durrant et al., 2014)	NetCDF	3.06; 5.96; 6.07; 11.22
CgE_{99th} (kW/m)	WaveWatchIII (Durrant et al., 2014)	NetCDF	8.35; 36.13; 35.45; 68.00
Tidal range (cm)	FES2014 model (Carrere et al., 2016; Lyard et al., 2016)	NetCDF	75.97; 177.43; 176.54; 206.65
Storms (n)	Number of storms recorded within 100km of islands between 1900 – 2017 (IBTrACS; Knapp et al., 2018)	Arc Shapefile	0.00; 20.24; 13.00; 145.00
Precipitation (m)	Monthly average precipitation - Copernicus Climate Change Service (C3S) (2017)	Google Earth Engine	0.12; 0.22; 0.22; 0.31
**Local island scale properties**			
Island size (ha)	Planform land area from earliest shoreline record (ha)	Shoreline data	0.06; 38.19; 4.25; 2148.01
Location* (Windward/Leeward)	Classifies islands based on its position on the atoll.	High-res imagery; ArcMap	
Reef width (m)	Width of the reef platform	High-res imagery; ArcMap	222.99; 976.55; 772.79; 4581.74
Ratio – Island width to reef width (IR ratio)	Ratio of island to reef platform width. Determines the occupancy of an island	High-res imagery; ArcMap	0.06; 0.26; 0.24; 0.86

	on the reef platform – a small island on a broad reef has a lower score		
Distance from reef edge (m)	Distance between the reef edge and the edge of vegetation on an island.	High-res imagery; ArcMap	35.48; 278.24; 242.59; 1338.38
Circularity Ratio	Circularity ratio of the island ($4\pi A/P^2$) – represents island shape	Shoreline data; ArcMap	0.03; 0.55; 0.58; 0.97
NDVI (Vegetation density)	Mean Normalised Difference Vegetation Index<br>-1 to +1 (Sparse to dense vegetation)	Landsat 8;<br>Google Earth Engine	-0.18; 0.53; 0.58; 0.83
Beach rock*	Exposed beach rock along the island shoreline – indicator of a historically dynamic shoreline; classified as: Absence / Presence	High-res satellite imagery; ArcMap	Presence (26.34%)
Anthropogenic Modification*	Degrees of anthropogenic modifications as observed from high resolution satellite imagery; classified as: High / Moderate / None	High-res satellite imagery; ArcMap	High (1.79%); Moderate (17.92%); None (80.29%)
Island type*	Classified island types - Type I to IV, according to Richmond (1992)	High-res satellite imagery; ArcMap	Type I (36.92%); II (13.98%); III (23.48%); IV (21.86%)

***categorical variables**

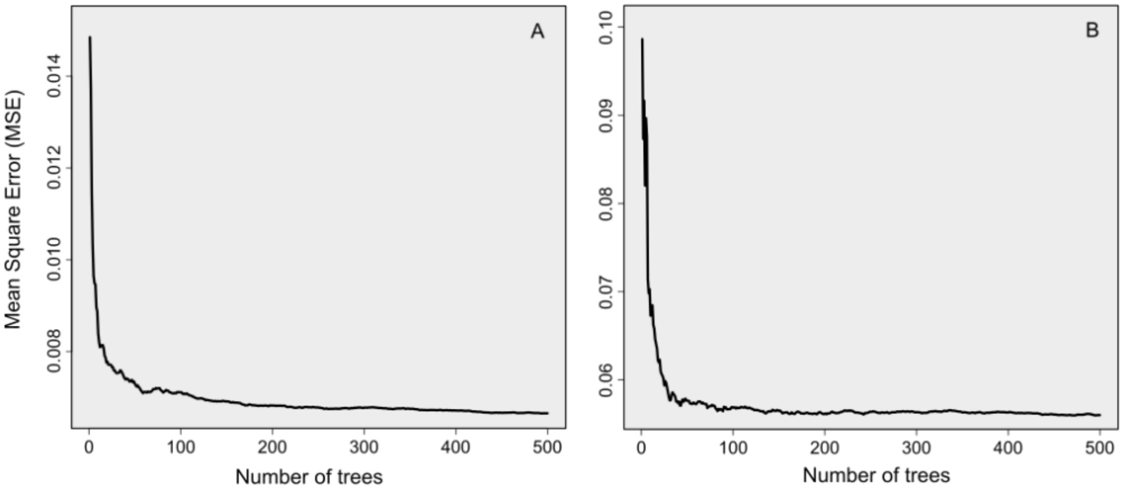

Supplementary Fig 2. Mean Square Error vs. the number of trees generated in the Random Forest models for response variables: (A) Island EPR and (B) Migration rate

Supplementary Table 2. Model evaluation metrics for the CART and Random Forest models developed for the response variables: shoreline change rate (Island EPR) and rate of migration

Response Variable	CART	Pruned CART	CART<br>10-fold cross validation	Random Forest	Random Forest<br>10-fold cross validation
Island EPR (m/decade)	R^2: 0.41<br>MAE: 0.51<br>RMSE: 0.69	R^2: 0.24<br>MAE: 0.56<br>RMSE: 0.79	R^2: 0.16<br>MAE: 0.65<br>RMSE: 0.87	R^2: 0.84<br>MAE: 0.24<br>RMSE: 0.36	R^2: 0.21<br>MAE: 0.57<br>RMSE: 0.80
Migration rate (m/decade)	R^2: 0.45<br>MAE: 1.14<br>RMSE: 1.85	R^2: 0.37<br>MAE: 1.23<br>RMSE: 1.97	R^2: 0.17<br>MAE: 1.46<br>RMSE: 2.43	R^2: 0.82<br>MAE: 0.61<br>RMSE: 1.05	R^2: 0.26<br>MAE: 1.34<br>RMSE: 2.10

Supplementary Table 3. Details of packages in R used for model development and evaluation.

R Package (version)	Details	Reference
**rpart**<br>**(4.1 – 15)**	Classification and Regression Tree (CART) models	Therneau and Atkinson (2018)

**randomForest (4.6 – 14)**	Random Forest models	Liaw and Wiener (2002)
**randomForestExplainer (0.10.1)**	Explanatory plots for random forest models, identify important predictors	Paluszynska et al. (2020)
**metrics (0.1.4)**	Evaluation metrics	Hamner et al. (2018)
**pdp (0.7.0)**	Partial dependence plots, examine variable interactions	Greenwell (2017)
**vip (0.2.2)**	Variable importance plots	Greenwell et al. (2018)

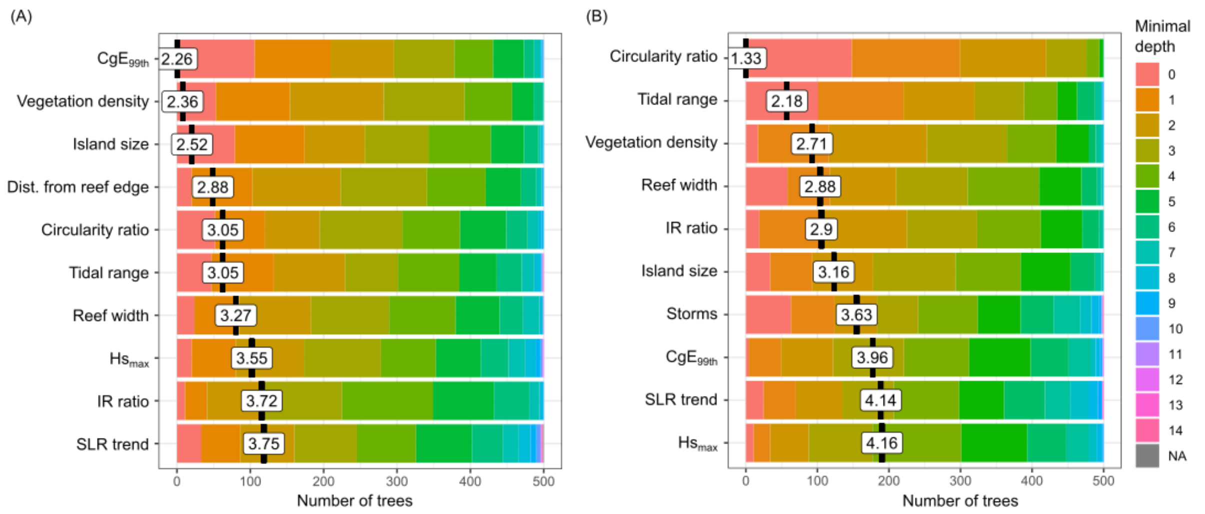

Supplementary Fig 3. Distribution of minimal depth and mean for Random Forest models for response variables (a) Island EPR (b) Migration rate

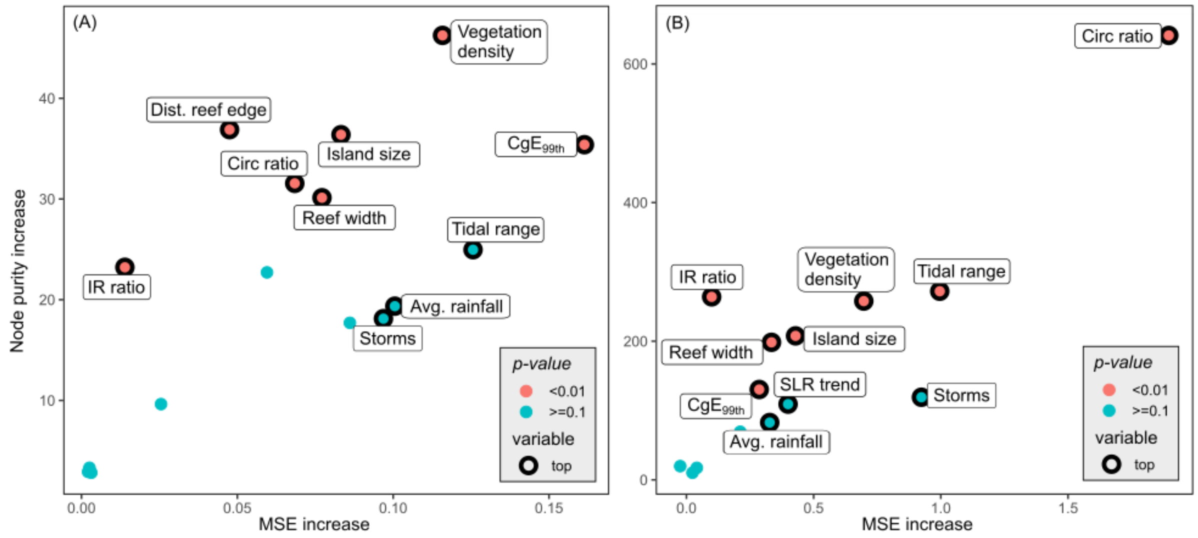

Supplementary Fig 4. Multi-way plots for relevant variables identified by the Random Forest models for response variables (a) Island EPR (b) Migration rate

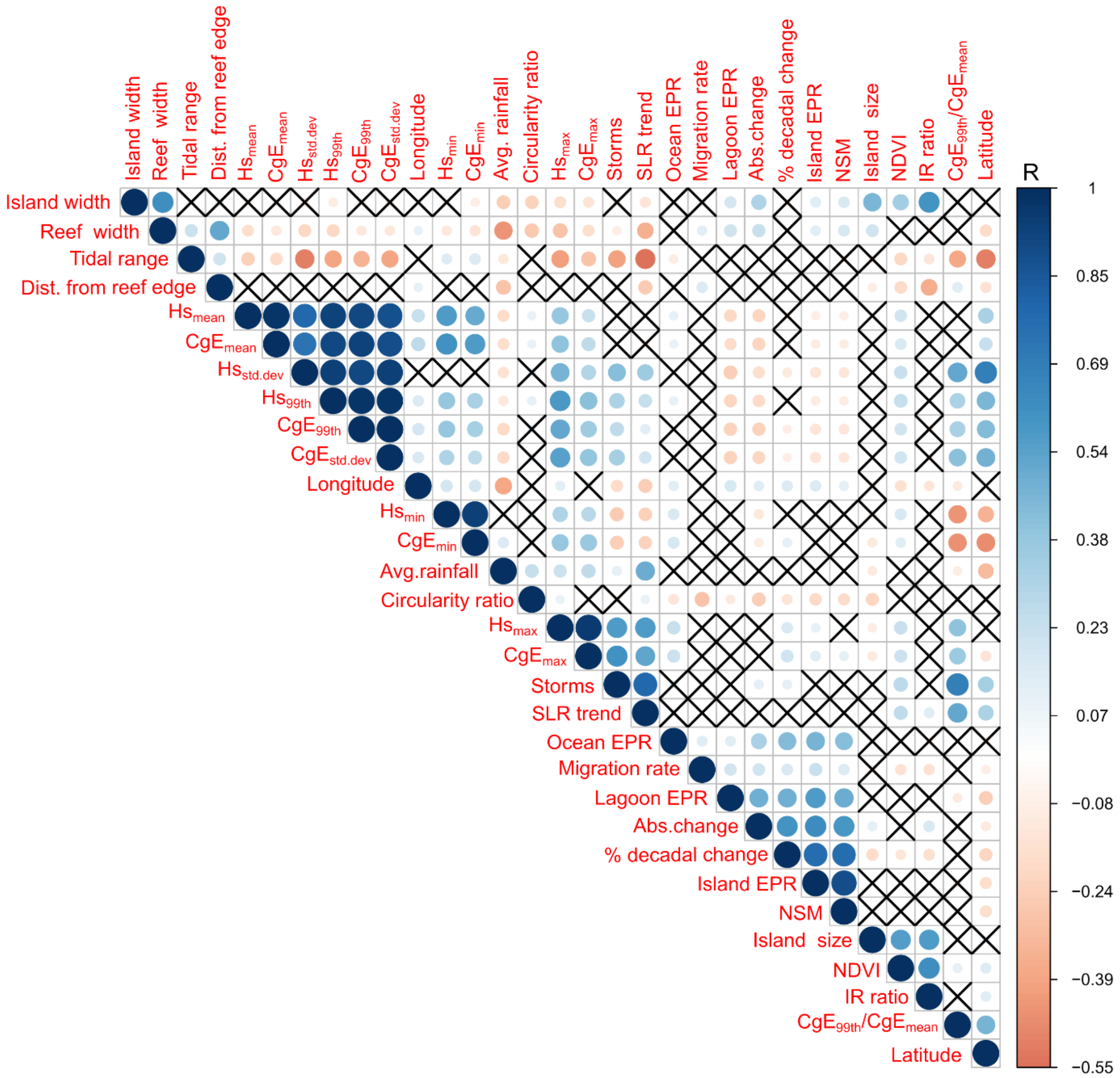

Supplementary Fig 5. Correlation plot showing strength of correlation (R) between all response and potential predictor variables (from Sengupta et al., 2023). Cross-marks indicate no significant correlation (95% confidence level). Response variables: Migration rate, Island EPR, Ocean and Lagoon EPR, absolute change in land area, % decadal rate of change in land area. Note that while $p < 0.05$ indicates statistical significance, this is due to sample size and the strength of correlation is weak ($R^2 < 0.1$)
